# Sentinel surveillance for influenza among severe acute respiratory infection and acute febrile illness inpatients at three hospitals in Ghana

**DOI:** 10.1111/irv.12397

**Published:** 2016-06-30

**Authors:** Alexander H. Jones, William Ampofo, Richard Akuffo, Brooke Doman, Christopher Duplessis, Joseph A. Amankwa, Charity Sarpong, Ken Sagoe, Prince Agbenohevi, Naiki Puplampu, George Armah, Kwadwo A. Koram, Edward O. Nyarko, Samuel Bel‐Nono, Erica L. Dueger

**Affiliations:** ^1^ Global Disease Detection and Response ProgramU.S. Naval Medical Research Unit No. 3CairoEgypt; ^2^ Noguchi Memorial Institute for Medical ResearchNational Influenza CenterAccraGhana; ^3^ Global Disease Detection and Response ProgramU.S. Naval Medical Research Unit No. 3 Ghana DetachmentAccraGhana; ^4^ U.S. Naval Medical Research Unit No. 3 Ghana DetachmentAccraGhana; ^5^ Ghana Health ServiceAccraGhana; ^6^ Tamale Teaching HospitalTamaleGhana; ^7^ 37 Military HospitalAccraGhana; ^8^ Noguchi Memorial Institute for Medical ResearchAccraGhana; ^9^ U.S. Centers for Disease Control & PreventionAtlantaGAUSA; ^10^ U.S. Naval Medical Research Unit No. 3CairoEgypt

**Keywords:** Ghana, human, influenza, sentinel surveillance

## Abstract

**Background:**

Influenza epidemiology in Africa is generally not well understood. Using syndrome definitions to screen patients for laboratory confirmation of infection is an established means to effectively conduct influenza surveillance.

**Methods:**

To compare influenza‐related epidemiologic data, from October 2010 through March 2013, we enrolled hospitalized severe acute respiratory infection (SARI; fever with respiratory symptoms) and acute febrile illness (AFI; fever without respiratory or other localizing symptoms) patients from three referral hospitals in Ghana. Demographic and epidemiologic data were obtained from enrolled patients after which nasopharyngeal and oropharyngeal swabs were collected, and processed by molecular methods for the presence of influenza viruses.

**Results:**

Of 730 SARI patients, 59 (8%) were influenza positive; of 543 AFI patients, 34 (6%) were positive for influenza. Both SARI and AFI surveillance yielded influenza A(H3N2) (3% versus 1%), A(H1N1)pdm09 (2% versus 1%), and influenza B (3% versus 4%) in similar proportions. Data from both syndromes show year‐round influenza transmission but with increased caseloads associated with the rainy seasons.

**Conclusions:**

As an appreciable percentage of influenza cases (37%) presented without defined respiratory symptoms, and thus met the AFI but not the SARI definition, it is important to consider broader screening criteria (i.e., AFI) to identify all laboratory‐confirmed influenza. The identified influenza transmission seasonality has important implications for the timing of related public health interventions.

## Introduction

The general dearth of influenza surveillance in Africa limits accurate understanding of influenza epidemiology,^1^, ^2^, ^3^ and in 2009, it contributed to the relatively late detection of the A(H1N1)pdm09 on the continent.^4^ Influenza remains a major contributor to global morbidity and mortality resulting in approximately 500 000 deaths annually.^5^ In West Africa, including Ghana, sentinel surveillance conducted in 2009–2010 detected influenza in 21–25% of samples from influenza‐like illness and 9% of severe acute respiratory infection (SARI) patients.^6^ With increasing diagnostic capacity and accelerated surveillance established throughout Africa in response to the 2009 pandemic, there is ample evidence that influenza is indeed prevalent, occurs throughout the year, and is culpable for an appreciable attributable burden of febrile illnesses.^4^ In line with the objectives of its National Preparedness and Response Plan for Avian and Pandemic Influenza: 2005–2006, the Ghana Health Service began influenza surveillance in 2007.

Surveillance using syndrome definitions to screen patients for enrollment in sentinel sites is an effective means to describe the epidemiology and etiologies of both respiratory and undifferentiated febrile illness and can contribute to the appropriate delivery of public health interventions.^7^, ^8^ Such surveillance for respiratory syndromes, including SARI, has been shown to effectively identify influenza cases,^9^ and several studies have also confirmed influenza infection among hospitalized and non‐hospitalized patients with undifferentiated febrile illness.^4^, ^7^ In young children, influenza patients often present for medical treatment with only fever;^10^ one recent study found that 19% of 106 influenza‐positive hospitalized infants <3 months old had fever but no respiratory symptoms.^11^From October 2010 and March 2013, we concurrently conducted surveillance for severe acute respiratory infection and acute febrile illness (AFI) in three referral hospitals in Ghana to compare influenza‐related epidemiologic data from the two syndrome‐based surveillance platforms.

## Methods

### Ethical considerations

The study protocol was approved by the institutional review boards of the Ghana Health Service, US Centers for Disease Control and Prevention, and Noguchi Memorial Institute for Medical Research. The study protocol was also approved by the US Naval Medical Research Unit No. 3 Institutional Review Board in compliance with all applicable Federal regulations governing the protection of human subjects. Written informed consent was obtained from all patients 18 years or older. Enrollment of patients under 18 years old required consent from a parent or guardian and assent from any child aged 5 < 18 years.

### Site selection and study enrollment

**Figure 1:**
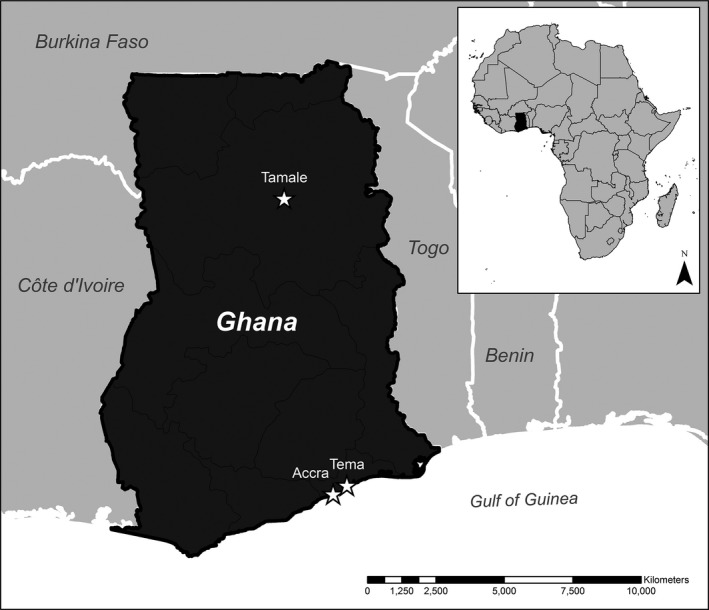
Ghana ‐ sentinel surveillance sites.

Hospitalized patients were enrolled at three major referral centers (Figure 1): Tamale Teaching Hospital in Ghana's Northern Region and Tema General Hospital and 37 Military Hospital both in the Greater Accra Region, southern Ghana. Serving both military and civilian patients, 37 Military Hospital is a teaching medical facility with 620 inpatient beds located in Accra. Also along Ghana's Atlantic coast, Tema General Hospital has 344 inpatient beds. Serving the Northern Region, Tamale Teaching Hospital can admit 320 patients. All sites participate in the National Health Insurance Scheme, which enables residents to obtain services without payment at the point of delivery.

Trained nurses and medical assistants conducted daily active surveillance by monitoring inpatient admission logs and then interviewing patients. After enrollment, study personnel collected risk factor and clinical information from patients using a standardized questionnaire stored on personal digital assistants. Trained laboratory staff collected biological specimens. Weekly, data were transferred from the devices to a central Microsoft Access^®^ database. At least weekly (before data transfer) supervisors reviewed data for validation and data cleaning purposes. Following data migration automated checks were used to rectify any data collection errors.

### Enrollment criteria

In SARI surveillance, we used multiple sets of enrollment criteria (Table 1) in an attempt to increase surveillance sensitivity for influenza. SARI case definitions for all potential patients included fever with respiratory symptoms. For SARI patients <5 years old, case definitions allowed for enrollment when patients presented with respiratory symptoms with or without fever. In AFI surveillance, we enrolled patients with a self‐reported history of “high” fever with the illness or documented current fever ≥38°C and no obvious source of localized infection; patients with malaria were included. Patients meeting the enrollment criteria for SARI were excluded from AFI enrollment. For both SARI and AFI surveillance, neonates (≤31 days of age) were not enrolled because of local concerns about collecting biological specimens from this age group.

**Table 1:** Severe acute respiratory* infection (SARI) and acute febrile illness (AFT) case definitions

Severe acute respiratory infection					Acute febrile illness, patients >31 days
Used for all patients >31 days			Only Used for Patients **>**31 days and <5 years		
2006 Adult SARI: Current fever ≥38°C AND Cough or sore throat AND Shortness of breath or difficulty breathing	CDC‐TFTP: Current Fever ≥38°C or hypothermia or abnormal white blood cell count AND One of the following: Tachypne Cough Abnormal breath Sounds Sputum Production Hemoptysis Chest pain or Dyspnea	2011 WHO SARI: Current fever ≥38°C or history of fever with this illness AND Cough With onset in the last 7 days	IMCI severe: Tachypnea, cough, or difficulty breathing AND One of the Following: Unable to drink breastfeed‐lethargi a unconsci ous Vomiting Convulsions Nasal flaring Grunting Oxygen saturation <90% Chest indrawing or Strides	IMCI moderate: Tachypnea AND Cough or difficulty breathing	History of fever during the course of this illness OR Current fever of 38°C Exclusion Criteria: Meets the SARI case definition Evidence of otitis media Evidence of septic arthritis Evidence of pyogenic soft tissue infection Evidence of urinary tract infection Evidence of cellulites Other obvious localised source of infection

CDC‐IEIP, US Centers for Disease Control and Prevention, International Emerging Infections Program; WHO SARI, World Health Organization severe acude respiratory infection; IMCI, Integrated Management of Childhood Illeness.

### Specimen collection and testing

Nasopharyngeal and oropharyngeal swabs were collected from all enrolled patients. At surveillance sites after collection, swabs were placed in viral transport medium (VTM), refrigerated, and within 48 hours, sent to the National Influenza Center for testing. In the laboratory, each vial was vortexed for 30 seconds and an aliquot of 200 μl of VTM transferred into a new vial for molecular testing and virus isolation of influenza‐positive samples. The original vial containing the swab and the remaining VTM were stored at −70°C. Stored VTM was processed for ribonucleic acid (RNA) extraction with the QIAmp viral RNA kit (Qiagen, Hilden, Germany). The final RNA extract was eluted in 60 μl of RNase/DNase‐free elution buffer from the QIAmp extraction kit. The sample was assayed with a real‐time reverse transcriptase polymerase chain reaction (rRT‐PCR) test for human influenza virus detection and characterization (rRT‐PCR Flu Panel) from the US Centers for Disease Control and Prevention.

### Data analysis

Data were analyzed using EpiInfo 7. Fisher's exact test (2‐tailed) was used to determine statistical significance of categorical variables. The Mann–Whitney–Wilcoxon two‐sample test, two‐tailed (for non‐normal distributions), was used to determine the statistical significance of medians. The Spearman's rank correlation test was used to determine association between monthly precipitation and caseloads. All *P*‐values were considered significant if ≤0·05.

## Results

Between October 2010 and March 2013, 730 SARI and 543 AFI patients were enrolled (Table 2). Demographic characteristics of all SARI and AFI patients were similar, with median ages of 12 (range 0·08–90) and 11 (range 0·08–99) years, respectively (*U* = 197 380; *P* > 0·99); median ages of SARI and AFI influenza cases were 7·0 (range 0·17–83) and 9·5 (range 0·75–47) years, respectively (U = 1003; *P* > 0·99). Gender was not significantly different between either SARI and AFI enrolled patients or SARI and AFI influenza cases. The predominant age group in SARI (45%) and AFI (39%) patients was 1 month – <5 years old.

**Table 2:** Demographic characteristics of enrolled patients for severe acute respiratory infection and acute febrile illness by viral influenza etiology, October 2010 – March 2013

Characteristic	Samples tested for influenza (*N* = 1273)							
	SARI (*n* = 730)				AFI (*n* = 543)			
	A(H1N1)p				A(H1N1)pd			
	A(H3N2)	dm09	Influenza B	Influenza negative	A(H3N2)	m09	Influenza B	Influenza negative
Age group (*n* = 727)					Age group (*n* = 543)			
<5 years old	12 (3·6)	9 (2·7)	7 (2·1)	302 (91·5)	1 (0·5)	1 (0·5)	6 (2·9)	202 (96·2)
6–18 years old	0 (0)	2 (2·9)	3 (4·4)	63 (92·6)	3 (2·9)	4 (3·9)	7 (6·9)	88 (86·3)
19–50 years old	3 (1·6)	4 (2·1)	4 (2·1)	177 (94·1)	2 (1·2)	2 (1·2)	8 (5·0)	149 (92·5)
50+ years old	6 (4·3)	2 (1·4)	7 (5·0)	126 (89·4)	0 (0)	0 (0)	0 (0)	70 (100)
Total	21 (2·9)	17 (2·4)	21 (2·9)	668 (91·9)	6 (1·1)	7 (1·3)	21 (3·9)	509 (93·7)
Gender (*n* = 716)					Gender (*n* = 537)			
Gender, Male	12 (3·1)	8 (2·1)	12 (3·1)	351 (91·6)	4 (1·4)	2 (0·7)	12 (4·3)	258 (93·5)

Outcome data were unavailable for 53% and 50% of SARI and AFI patients, respectively. Collected outcomes covered patient discharges, absconders, transfers, and deaths. Almost half of missing outcomes were from patients <5 years old. Although 26 (2%) deaths were recorded among all enrolled patients (*N* = 1273), none were among SARI or AFI influenza cases.

Of all enrolled patients, 93 (7%) were positive for influenza. Among influenza‐positive cases, virus characterization revealed 51 cases of influenza A (55%) (A(H1N1) 26%; A(H3N2) 29%) and 42 cases of influenza B (45%) (Table 1). Influenza positivity rates among SARI (51/730, 8%) and AFI (34/543, 6%) patients were similar (*P* = 0·23). Patients older than five years constituted 31 (31/59, 53%) SARI influenza cases and 26 (26/34, 77%) AFI influenza cases.

Although influenza A(H3N2), A(H1N1)pdm09, and influenza B were detected in both SARI and AFI patients, no patients were positive for seasonal A(H1N1) or A(H5N1). Circulating influenza subtypes were similar in SARI and AFI patients, but there was a significantly higher proportion of influenza A cases among SARI patients (38/730, 5%) than in AFI patients (13/543, 2%, *P* = 0·01). Also, influenza A(H3N2) was more common among SARI patients (3%) than AFI patients (1%, *P* = 0·03). There was no significant difference in the proportions of other subtypes between patients with the two syndromes.

Among SARI influenza cases, 58 (58/59, 98%) presented with current or history of fever and cough. Abnormal breath sounds (42%), chest pain (34%), and sputum production (29%) were also common among SARI influenza cases. Per the case definition, AFI influenza cases presented with current or history of fever but no known source of infection.

**Figure 2:**
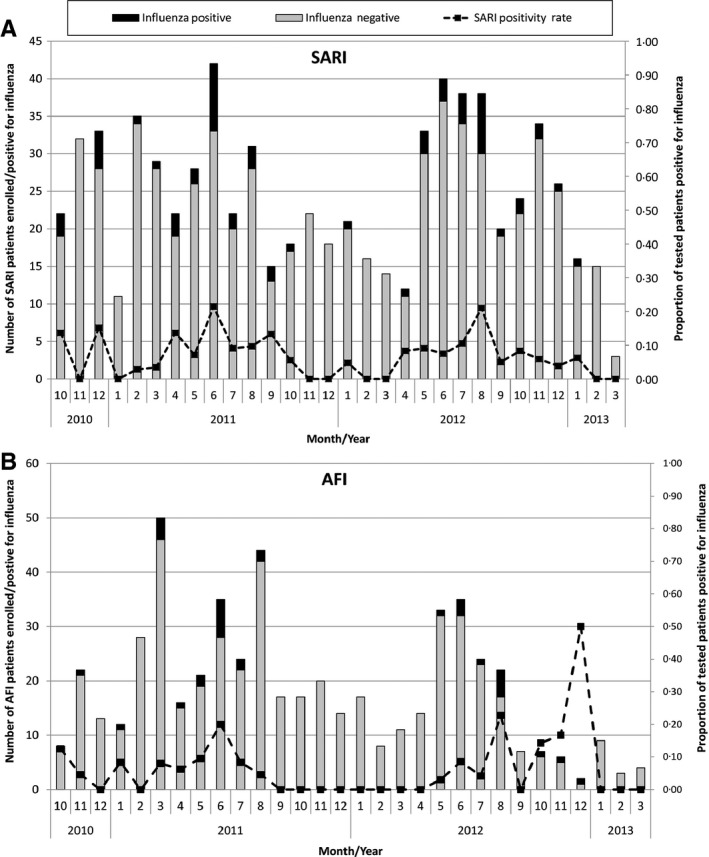
A ‐ Number of severe acute respiratory infection patients tested for influenza (*N* = 730) and results by month. B ‐ Number of acute febrile illness patients tested for influenza (*N* = 543) and results by month. October 2010 – March 2013.

**Figure 3:**
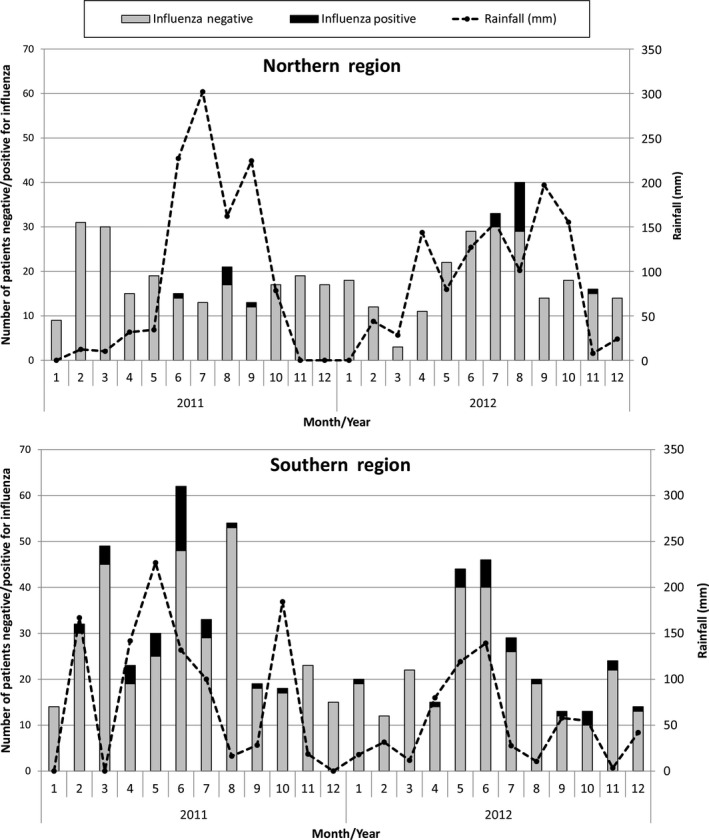
A – Northern region, number of patients tested for influenza (*N* = 449) and results by month. B – Southern region, number of patients tested for influenza (*N* = 644) and results by month. January 2011‐December 2012.

A comparison of 2011 and 2012 data shows SARI and AFI influenza cases have similar temporal distributions with cases peaking in June and August of each year (Figure 2). Positivity rates showed peaks in June 2011 and August 2012. During the 2011 ‐ 2012 calendar years, the surveillance site in northern Ghana reported four (4/6, 67%) of its annual influenza cases in August 2011 and 11 (11/15, 73%) in August 2012 (Figure 3). The two sites in southern Ghana reported peak annual influenza cases (14/36, 39%) in June 2011 and June 2012 (6/23, 26%). During the study period, the southern sites had a significantly higher influenza positivity rate (71/756, 9%) than the northern site (22/517, 4%; *P* < 0·001). Statistical testing showed that for all influenza cases, the timing of case peaks and rainfall, as reported by the Ghana Meteorological Agency, had a positive correlation (*r*_s_ = 0·50, *P* = 0·01).

## Discussion

During the study period, influenza regularly contributed to SARI and AFI among hospitalized patients in Ghana. Accurate determination of influenza epidemiology to support public health policy decisions is challenging, especially in countries relying solely on sentinel site surveillance data. Utilization of SARI case definitions alone for influenza surveillance, as is common practice, may substantially underestimate the number of hospitalized influenza cases because influenza‐positive febrile cases often present with unidentified or non‐respiratory symptoms.

In our study, with concurrent active SARI and AFI surveillance for 30 months, influenza in both SARI and AFI patients indicated influenza B (42/93, 45%) as the dominant influenza type in hospitalized patients in Ghana. During the study period, the World Health Organization reported the same influenza types and subtypes circulating in sub‐Saharan Africa^12^ as our surveillance detected. From our study, the similar circulating influenza types and subtypes among SARI and AFI patients show that the clinical signs and symptoms resulting from specific types and subtypes often overlap. Thus, our findings suggest SARI surveillance alone may demonstrate the influenza type and subtypes circulating in a given place during a given time period.

As almost all SARI influenza cases (98%) presented with (current or history of) fever and cough, a simplified SARI case definition would not have greatly reduced surveillance sensitivity for influenza within that syndrome. Other respiratory symptoms were present but less common among SARI influenza cases. Additional research can help inform decision‐making on a simplified SARI case definition for influenza surveillance.

The substantial proportion (34/93, 37%) of influenza cases among hospitalized patients in Ghana detected using AFI surveillance and thus presenting without or with unidentified respiratory symptoms is notable. One 2009 sentinel surveillance study from Puerto Rico recorded 48% of hospitalized AFI patients without respiratory symptoms were influenza positive.^13^ The reasons for variations in the proportion of influenza cases without or with unidentified respiratory symptoms among febrile patients may depend on study design and implementation. Regardless, in Ghana, a substantial proportion of hospitalized influenza patients present with difficult to detect or lacking respiratory symptoms.

Notably, the majority (77%) of AFI influenza cases were older than five years. The aforementioned Puerto Rico study also found the majority of detected influenza cases among hospitalized and outpatient AFI patients were older than five years.^13^ Other studies have detailed a lack of respiratory symptoms among influenza cases, but those findings were primarily among children. In 2002, a study in France found 30% of influenza‐positive inpatient and outpatient children had minimal or no respiratory symptoms.^10^ Although we did not enroll neonates in our study, Bender *et al*.^11^showed that hospitalized and outpatient influenza cases in the United States <3 months of age often present with only fever.

Among SARI and AFI patients in our study, the timing of peak influenza caseloads and specific types and subtypes suggest possible year‐long transmission with seasonal peaks (Figure 2) although further surveillance is required. Seasonality in influenza transmission is consistent with findings from other tropical countries^1^, ^14^, ^15^, ^16^ including Tanzania^17^ and Brazil.^18^ In Senegal, influenza peaks also generally correspond to months with higher precipitation and humidity.^19^, ^20^

As in Senegal and Brazil, seasonal influenza peaks generally correspond with the two rainy seasons in Ghana: May–June and August–September. Interestingly, the timing of precipitation (and corresponding increased humidity) and influenza case peaks correspond to regional climatic differences; southern Ghana experiences earlier case and precipitation peaks than northern Ghana. As our southern Ghana surveillance sites are closer to the equator than the northern site, the timing of peak influenza caseloads is similar to patterns observed in Brazil where influenza transmission occurs earlier close to the equator and moves later in the year away from the equator.^18^

In French Guiana, Mahamat et al.^21^ demonstrate influenza seasonality associated with increased rainfall. Additionally, a review from sub‐Saharan African nations suggests influenza seasonality, aligning with the months of June–August and with timing generally corresponding to distance from the equator.^22^ Influenza prevalence exhibits seasonality coinciding with the rainy season in Cote d' Ivoire.^23^ Influenza diagnosed in children <11 years of age in Ghana exhibits slight seasonality during the months of May through July, again coinciding with the rainy season.^24^

Our study has several limitations. The number of detected influenza cases was relatively small restricting our ability to conduct statistical analyses and observations. We did not enroll neonates in this study, potentially resulting in substantial underestimation of influenza cases in young children. After enrollment, we did not follow AFI patients to determine whether they developed respiratory symptoms. The 30‐month study period consisted of two rainy season in 2011 and two in 2012. This limited our ability to fully examine seasonality and define a flu season in Ghana. Also, we did not collect data on host behaviors, including during the rainy season; such data could improve understanding of specifically how seasonality affects influenza transmission. Additional data on humidity and precipitation would allow for a clearer understanding of the link between influenza transmission and those climatic factors. Finally, missing outcome data reduced our ability to investigate disease severity among the study population.

Concurrent SARI and AFI surveillance revealed similar epidemiological pictures of influenza among hospitalized patients in Ghana in terms of demographics, (sub‐)type distribution, and seasonality. However, the use of AFI surveillance highlighted a substantial underestimation of the number of influenza cases among the study population compared to SARI surveillance alone. Costs of additional surveillance efforts, even for a relatively short time period, may be justified when trying to improve understanding of influenza epidemiology to support public health interventions, such as vaccination and health education campaigns. Additionally, the evidence of seasonality in influenza transmission may inform the timing of potential influenza vaccination campaigns (and other interventions) in Ghana. Although these surveillance data provide a baseline for understanding the complexities of influenza epidemiology in Ghana, further research is warranted to explore influenza seasonality by region, disease severity and associated risk factors, and disease incidence in order to adequately inform public health policy decisions.

## Funding

This work was supported by the U.S. Centers for Disease Control and Prevention [Protocol #908] and the Global Emerging Infections Surveillance and Response System (GEIS) [Grant: C0236_10_N3] Operations, a division of the Armed Forces Health Surveillance Center (AFHSC).

## Disclaimer

The views expressed in this work are those of the authors and do not represent the official policy of the US Government, the US Centers for Disease Control and Prevention, the Department of Defense, or the Department of the Navy. The study protocol # NAMRU3·2009·0008 entitled “Integrated Hospital‐Based Infectious Disease Surveillance in the Greater Accra and Northern Regions, Ghana” was reviewed and approved by the institutional review boards of the US Naval Medical Research Nit No. 3, Noguchi Memorial Institute for Medical Research, Ghana Health Service, and US Centers for Disease Control and Prevention in compliance with all federal regulations governing the protection of human subjects.
